# Improving RLRN Image Splicing Detection with the Use of PCA and Kernel PCA

**DOI:** 10.1155/2014/606570

**Published:** 2014-09-14

**Authors:** Zahra Moghaddasi, Hamid A. Jalab, Rafidah Md Noor, Saeed Aghabozorgi

**Affiliations:** Faculty of Computer Science and Information Technology, University of Malaya, 50603 Kuala Lumpur, Malaysia

## Abstract

Digital image forgery is becoming easier to perform because of the rapid development of various manipulation tools. Image splicing is one of the most prevalent techniques. Digital images had lost their trustability, and researches have exerted considerable effort to regain such trustability by focusing mostly on algorithms. However, most of the proposed algorithms are incapable of handling high dimensionality and redundancy in the extracted features. Moreover, existing algorithms are limited by high computational time. This study focuses on improving one of the image splicing detection algorithms, that is, the run length run number algorithm (RLRN), by applying two dimension reduction methods, namely, principal component analysis (PCA) and kernel PCA. Support vector machine is used to distinguish between authentic and spliced images. Results show that kernel PCA is a nonlinear dimension reduction method that has the best effect on R, G, B, and Y channels and gray-scale images.

## 1. Introduction

For many decades, photography has served a vital function in the lives of people and has been considered as one of the most important revolutions in recording moments. However, photographs have recently lost their trust because of the increasing use of manipulation tools, which have made photographs less trustable. The history of image tampering (i.e., creating photographs that never really happened in real life) is as old as the art of photography itself. Images have been applied for malicious purposes in many instances. In 2003, the Los Angeles Times printed on its front cover an image from photojournalist, Brian Walski, which showed a British soldier in Iraq trying to control a crowd of civilians in a passionate manner. However, the depicted moment never happened. The photograph was a composite of two different photographs merged to create an appealing image. The image tampering was discovered, and Walski was fired [1]. In 2009, the Brazilian newspaper Folha de Sao Paulo published an article that contained a spliced image created from different gray-scale images to show how the Brazilian Chief of Staff actively participated in the resistance against the military regime through such actions as the planning of and preparation for robberies and kidnappings [1].

An innovative research area called digital image forensics emerged from the necessity to regain the trustability of photographs. Digital image forensics is generally categorized into two basic groups: active methods and passive methods [2]. Active methods borrow additional information to insert into the original image, whereas passive methods require no prior information. Digital signatures and watermarking are the most common active detection methods. Passive methods focus on statistical modifications made on image content. Such modifications include splicing, retouching, healing, copy-move, and blurring. Image splicing is best defined as a combination of two or more parts of different photographs to create a new image, which is known as a spliced image. Image splicing deceives the human visual system to achieve its malicious purposes.

Studies were conducted on this topic, and several image splicing detection methods were proposed and developed. Farid [3] considered speech signal splicing as a highly nonlinear process and applied higher order spectral analysis to solve this problem. Ng et al. [4] extended the previously mentioned scheme into an image splicing detection method with the assumption that the image splicing procedure is nonlinear and that the image involved is nonstationary. Their expanded method achieved an unsatisfactory detection performance of 72%.

Fu et al. [5] proposed an image splicing detection approach using the Hilbert-Huang transform (HHT). They considered the high nonlinearity and nonstationary nature of the image splicing operation and merged this technique with a statistical natural image model on the basis of moments of characteristic functions with wavelet decomposition. Their method obtained an accuracy rate of 80.15%. Li et al. [6] combined two methods, namely, moment features based on first-order histogram of the image discrete wavelet transform (DWT) coefficients and HHT, to extract features for image splicing detection. Their method obtained an average accuracy rate of 85.87%.

Shi et al. [7] proposed a blind, passive, and natural image splicing detection model based on statistical feature extraction methods. Their model includes the Markov transition probabilities and moments of characteristic functions of wavelet subbands applied to different 2D arrays and to the 2D arrays of multisize block discrete cosine transform. Their model achieved a detection accuracy rate of 91.8%, which is a promising improvement in the image splicing detection area.

Zhang et al. [8] applied an idea in image steganalysis [9] by merging the Markov features and discrete cosine transform (DCT) features. They achieved a detection rate of 91.5%. He et al. [10] expanded the natural Markov-based model applied in [7] by capturing the interblock correlation between the block DCT coefficients proposed in [11] and merging it with the features generated from the DWT domain. They also reduced their features by applying a feature selection method called support vector machine recursive feature elimination (SVM-RFE), which achieved a detection rate of 93.55% on a digital video multimedia (DVMM) image dataset [12].

Dong et al. [13] proposed an approach based on statistical features obtained from the run length method and on image edge statistics from the blind image splicing detection method. He et al. [14] improved the method proposed in [13] and developed a detection algorithm based on approximate run length. Their results showed a moderate detection accuracy rate (75% versus 69%) but with a lesser amount of time than in the original algorithm (6D versus 12D). Zhao et al. [15] proposed applying the run length run number (RLRN) vectors in four directions of the chroma spaces because detecting spliced images in one color space is difficult. Their results showed that the features extracted from the chroma channels were more accurate than those extracted from the R, G, B, and Y channels.

The existing methods concentrate only on the actual image splicing detection techniques, and handling the extracted high dimensional and redundant features can be a difficult and time consuming process. Therefore, dimensionality reduction methods are applied to remove redundant information from the extracted features and to obtain the most discriminative information with less dimensionality.

In this paper, we compared the two dimension reduction methods (PCA versus kernel PCA) that were developed. This paper aims to evaluate the detection accuracy and computational time of PCA and kernel PCA on RLRN [15] in dimensionalities of 10 and 50. The rest of this paper is organized as follows. The proposed approach is described in the second section, the experimental results are discussed in the third section, and the conclusion and future works are presented in Section 4.

## 2. The Proposed Approach

**Figure 1:**
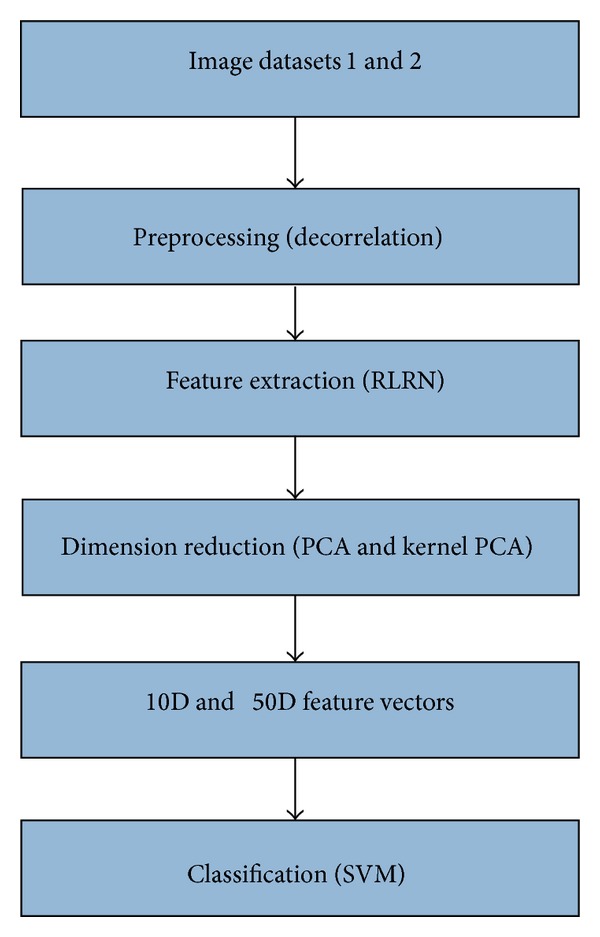
Proposed algorithm framework.

Figure 1 demonstrates the approach proposed in this paper. The approach includes four phases: preprocessing, feature extraction, dimension reduction, and classification. The given image from image dataset 1 [12] or image dataset 2 [16] is initially preprocessed for extraction. The features are then extracted from the preprocessed image using RLRN [15]. The dimensionality is then reduced to 10 and 50 dimensions by applying PCA and kernel PCA. Finally, the *n* dimensional (*n*D) feature vector is used as input for the support vector machine (SVM) classifier to calculate the detection accuracy and computational time.

### 2.1. Preprocessing

Preprocessing improves image content by reducing undesired distortions and/or enhancing image features relevant to further processing. A difference between 2D arrays is used to reduce the correlation between image pixels/coefficients and the effect caused by the diversity in image content [15, 17]. The difference between 2D arrays is computed as follows [15, 17]:

(2.1) $$\begin{array}{c} E_{h}\left(i,j\right)=x\left(i,j\right)-x\left(i+1,j\right)\\ E_{v}\left(i,j\right)=x\left(i,j\right)-x\left(i,j+1\right)\\ E_{d}\left(i,j\right)=x\left(i,j\right)-x\left(i+1,j+1\right)\\ E_{m}\left(i,j\right)=x\left(i+1,j\right)-x\left(i,j+1\right), \end{array}$$

where *x*(*i*, *j*) represents the *i*th and *j*th element of the gray value of the images in the image matrix and *E*_*h*_(*i*, *j*), *E*_*v*_(*i*, *j*), *E*_*d*_(*i*, *j*), and *E*_*m*_(*i*, *j*) represent the elements along the horizontal, vertical, diagonal, and minor-diagonal directions, respectively.

### 2.2. Feature Extraction Methods

Image features include the global and local properties of an image such as average gray levels, intensity histogram shapes, circles, lines, texels, and contour shapes. Different methods have been developed to extract these features, and these methods have been applied in various image processing fields. Given that image splicing detection is a two-class problem (i.e., one class for authentic images and another class for spliced images), features extracted from images serve an important function in the detection and classification process, which aims to distinguish authentic images from spliced ones. In this paper, a recently used feature extraction method is presented to examine the effect of dimension reduction techniques on detection performance.

#### 2.2.1. RLRN

RLRN is not extensively applied as a feature extraction method. However, the results which have been obtained in [15] show that this method can be used as an image splicing detection approach. The method was initially used in [18] in texture analysis to classify a set of terrain samples. A new run length algorithm was developed in [19] to extract texture features based on a multilevel dominant eigenvector estimation method, which improves classification accuracy.

This paper applied RLRN and its definition and mathematical equations are presented in this section. Zhao et al. [15] suggested using this method. A run in an image is defined as the number of pixels with the same gray level value in a specific direction. For a given image, a run length matrix *p*_*θ*_(*i*, *j*) is defined as the number of runs with gray level *i* and run length *j* along a specific direction. Hence, the run length vector is defined as follows [15]:

(2) $$\begin{array}{c} p_{\theta r}\left(j\right)=\overset{M}{\underset{i=1}{\sum }}p_{\theta }\left(i,j\right)\;1\leq j\leq N, \end{array}$$

where *M* represents the number of gray levels and *N* is the maximum value of run lengths. Vector *p*_*θr*_(*j*) demonstrates the sum distribution of runs with length *j* in a given image. In the equation, run length represents the spread of the image structure and texture. The image with a long run length is smoother than that with a short run length because the latter has different regions with different structures. The gray level run length pixel number matrix is used to equally emphasize all run lengths in this paper. The matrix is defined as follows [15, 19]:

(3) $$\begin{array}{c} p_{\theta pr}\left(j\right)=\;\overset{M}{\underset{i=1}{\sum }}p_{\theta p}\left(i,j\right)\;1\leq j\leq N, \end{array}$$

where

(4) $$\begin{array}{c} p_{\theta p}\left(i,j\right)=p_{\theta }\left(i,j\right)\cdot j, \end{array}$$

where *p*_*θpr*_(*j*) is the feature vector applied and referred to as RLRN. Four RLRN vectors were captured in four directions (0°, 45°, 90°, and 135°) from every channel (R, G, B, Y, Cb, and Cr) in image dataset 2 and from gray-scale images in image dataset 1 to distinguish the spliced images from the authentic images. The RLRN feature vector is shown as a 100D feature vector by obtaining the first 25 features of every vector in each orientation (25 × 4 = 100).

The run length of an image represents its structure and texture, and the splicing procedure modifies the pixel correlations and image structure. Therefore, the RLRN feature extraction method can represent discontinuities and nonconformity and can be efficiently used as an image splicing detection method [13, 15].

### 2.3. Dimension Reduction Methods

Dimension reduction methods are applied to reduce feature dimensionality by eliminating redundant features and keeping important dimensions in the feature vector. Humans and machine learning methods find it difficult to interpret high dimensional data. Given that a feature matrix has rows that each represents a specific instance of an object and a large number of features exponentially increase the computational time. Thus, decreasing information into smaller sizes enhances method analysis and improves the training and testing phases during classification [20]. Several experiments were conducted to test and analyze this idea.

Different approaches such as finding the linear or nonlinear manifold that lies within the high dimensional data space can simplify interpretation. In this section, the PCA and kernel PCA are presented to improve the features extracted by the RLRN feature extraction technique discussed in the previous section. Moreover, linearity (PCA) and nonlinearity (kernel PCA) were considered in selecting the dimension reduction techniques to investigate their effects on the extracted features.

#### 2.3.1. PCA

PCA is the most common and popular linear dimension reduction approach [21–23]. It has been used for years because of its conceptual simplicity and computation efficiency. The approach is applied in many areas such as noise reduction, pattern recognition, regression estimation, and image indexing [24]. It maps a dataset of *n* dimensions to a linear subspace with *d* dimensions, where *d* < *n*, and attempts to maintain most of the variability in the mapped dimensions. PCA is considered a second-order approach depending on the covariance matrix of the variables. The approach has different names in different fields such as singular value decomposition, Karhunen-Love transform, Hotelling transform, and empirical orthogonal function method [25].

PCA is based on finding the *d* orthogonal linear vectors, known as principal components, of *n* dimensions with maximum variance. Therefore, the number of reduced dimensions is not more than *n*. The approach works well if the most significant modes of variability are almost linear. Hence, high dimensional samples are best remade from their low dimensional linear projections. Otherwise, PCA becomes ineffective if the most vital significant modes of variability are nonlinear [21]. In mathematical terms, PCA finds *Y* as the new feature vector set with *d* dimension (*d* ≤ *D*), in which *X* is the original feature vector set with *D* dimension [26]:

(5) $$\begin{array}{c} Y=XM. \end{array}$$

To find linear mapping* M*, PCA attempts to maximize the following function:

(6) $$\begin{array}{c} M^{T}\mathrm{cov}\left(X\right)M, \end{array}$$

where cov⁡(*X*) is the covariance of the original feature vector set *X*. However, *M* consists of *d* principal eigenvectors of the sample covariance matrix of the zero-mean data [26]. Therefore, the following eigenproblem must be solved for the *d* principal components *λ*:

(7) $$\begin{array}{c} \mathrm{cov}\left(X\right)M=\lambda M. \end{array}$$

#### 2.3.2. Kernel PCA

PCA is a linear dimension reduction method. Some datasets have a nonlinear nature, and PCA cannot reduce the dimensions of these datasets efficiently. Thus, kernel PCA was designed to address this problem. Kernel PCA was applied in some pattern recognition experiments [24] and exhibited better recognition rates than linear PCA. Kernel PCA is a nonlinear form of PCA that attempts to identify complicated correlations between given features. It computes principal components in the original dataset through nonlinear mapping. It also discovers major components that are nonlinear in relation to the input space by running, which results from nonlinear mapping in which the low dimensional hidden structures are likely to be simple [21]. Kernel PCA locates the principal eigenvectors of the kernel matrix instead of the covariance matrix [26]. Thus, the computational complexity of kernel PCA is independent of the dimensionality of the feature set, which allows it to work on feature sets with different possible dimensionalities. Kernel PCA does not require any nonlinear optimization; it only needs to solve an eigenvalue problem as in the case of standard PCA. Thus, kernel PCA is free of local minima trap during training. The original feature set must be mapped to a higher dimensional feature set to calculate kernel PCA [24]:

(8) $$\begin{array}{c} \Phi :R^{N}⟶F,\;x⟶X. \end{array}$$

Then, the covariance matrix of data is calculated to obtain the principal components by solving the eigenvalue problem using the following equations:

(9) $$\begin{array}{c} C_{F}=\frac{1}{N}\overset{N}{\underset{1}{\sum }}\Phi \left(x_{i}\right)\Phi {\left(x_{i}\right)}^{T}\\ C_{F}v=\lambda v. \end{array}$$

Subsequently, the eigenvector can be expressed as a linear combination of features:

(10) $$\begin{array}{c} v=\overset{N}{\underset{1}{\sum }}\alpha_{i}\Phi \left(x_{i}\right)\;\\ \alpha_{i}=\frac{1}{\lambda N}v. \end{array}$$

Therefore, the kernel matrix is defined as follows:

(11) $$\begin{array}{c} k_{ij}=K\left(x_{i},x_{j}\right)=\left(\Phi \left(x_{i}\right)\cdot \Phi \left(x_{j}\right)\right)=\Phi {\left(x_{i}\right)}^{T}\Phi \left(x_{j}\right), \end{array}$$

where *k*_*ij*_ represents the elements of kernel matrix *K*, *x* is the feature set, and *K* is the kernel function with conditions that result in a positive semidefinite kernel *K* [26]. Φ(*x*_*i*_) may not be zero-mean such that the features must be centered. The corresponding kernel is obtained using the following equation:

(12) $$\begin{array}{c} k_{ij}^{c}=k_{ij}-\frac{1}{N}\overset{N}{\underset{i=1}{\sum }}k_{ik}-\frac{1}{N}\overset{N}{\underset{j=1}{\sum }}k_{jk}+\frac{1}{N^{2}}\overset{N}{\underset{i,k}{\sum }}k_{ik}. \end{array}$$

Consequently, the following equation represents the low dimensional feature set *y*_*i*_:

(13) $$\begin{array}{c} y_{i}=\overset{N}{\underset{i=1}{\sum }}\alpha_{ji}K\left(x,x_{i}\right), \end{array}$$

where *α*_*ji*_ represents the *j*th value in the vector *α*_*i*_.

**Figure 2:**
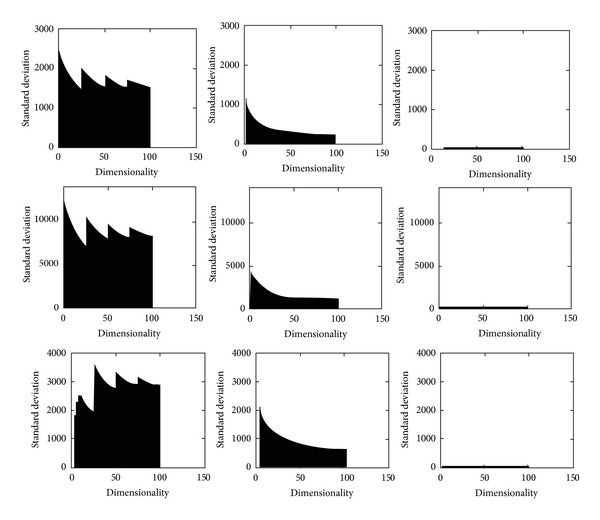
Standard deviation distributions of the extracted features. The rows indicate the standard deviation distributions of the features extracted from gray-scale images, red channel, and Cb channel of the colored images, respectively. The first column indicates the original features. The second column shows the features after applying PCA, while the third column shows the features after applying kernel PCA.

High correlations are generally found among the extracted features using RLRN. PCA and kernel PCA are applied to reduce the correlations by eliminating the information redundancies from the features.  Figure 2 shows the standard deviation distribution of the features extracted from gray-scale images (R and Cb channels of the colored images) before and after applying PCA and kernel PCA, respectively. The standard deviation measures and shows how data are spread out from the mean. In this case, a high standard deviation implies a high correlation between the features.  Figure 2 shows that the original features are highly correlated and their standard deviations are spread over a wide range in 100D. After applying PCA, the standard deviations mostly concentrate on the first few features and decrease as dimensionality increases. However, the standard deviations are still high and considerable. In contrast, the standard deviations greatly reduced after applying kernel PCA on the original features. The features after applying kernel PCA were obviously highly uncorrelated.

### 2.4. Classifier

SVM is one of the most popular supervised machine learning algorithms applied in pattern recognition. The Matlab codes for this classifier are available in [27]. In this work, LIBSVM was specifically used as a classifier, while radial basis function was used as a kernel function. A grid search method was applied to obtain the best value for the *c* and *g* parameters. All authentic images were labeled as −1 and all spliced images were labeled as +1 during the classification process.

## 3. Experimental Results

A set of experiments that demonstrate the effectiveness of the proposed approach are described. Our classification system was implemented using MATLAB R2012a on a 2.40 GHz Intel (R) Core i5 processor with 4 GB RAM on a Windows 7 platform.

### 3.1. Image Dataset

Two image datasets (gray and colored) were applied to evaluate the proposed method. The first image dataset was the Columbia Image Splicing Detection Evaluation Dataset provided by the Digital Video MultiMedia (DVMM) Laboratory, Columbia University (2007) [12]. This dataset contained 1845 gray-scale images (933 authentic images and 912 spliced images) in BMP format. DVMM was the only gray-scale image dataset designed for image splicing detection evaluation. Almost all papers applied DVMM, so it was also used in this work for better comparison with other methods. Another image dataset designed by the Chinese Academy of Sciences, Institute of Automation (CASIA), with high resolution images was also applied.

**Figure 3:**
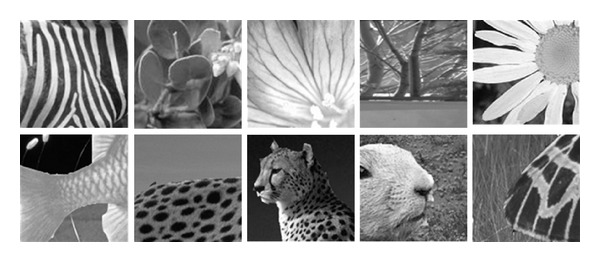
Examples of DVMM image dataset.

**Figure 4:**
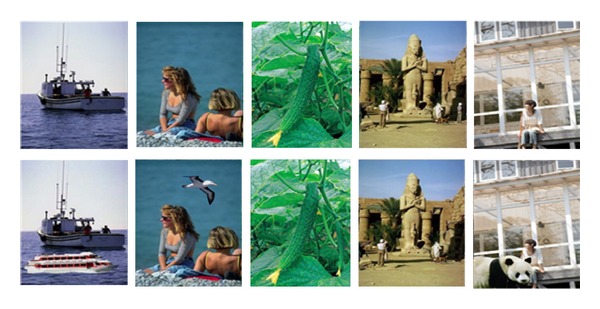
Examples of CASIA image dataset.

The CASIA tampered image detection evaluation database [16] is another image dataset designed to evaluate image splicing detection methods. Version 1.0 of this dataset included 1721 color images (800 authentic images and 921 spliced images) with 384 pixels × 256 pixels in JPEG format and was used in our experiments to evaluate the proposed approach. Examples of both image datasets are presented in Figure 3 (DVMM image dataset) and Figure 4 (CASIA image dataset). The first row consists of authentic images, while the second row consists of spliced images [12, 16]. Each image dataset is divided into two groups during the classification process: training (5/6 of the images) and testing (1/6 of the images). These groups were randomly selected to reduce the nondeterministic properties of the classifier.

### 3.2. Classification

For satisfactory results, PCA and kernel PCA were applied on RLRN feature extraction method with different dimensionalities (10D and 50D) to evaluate the detection accuracy and computational time. The results were presented in three tables. The true positive (TP) and true negative (TN) represent the detection rate of the authentic and spliced images, respectively. Accuracy (Acc.) represents the average detection rate. Computational time is represented in seconds (s) in each table.

Table 1 illustrates the results from the original dimension of the RLRN method with 100D obtained from different channels (RGB, gray, and YCbCr). The results show that the detection accuracies in the R, G, and B channels varied in a close range (64.66% to 69.30%), which verifies the strong correlation among the three channels because of the color filter array interpolation process. The luma channel (Y) that is correlated with the RGB channel (i.e., Y is a linear combination of R, G, and B channels) [15] also shows similar results (63.09%) with RGB. The results obtained from the Cb and Cr channels exhibited the best detection accuracies (89.59% and 93.80%) among the channels, since RLRN is more sensitive to the chroma channels (Cb and Cr) than the luma one [15]. However, the computational times for the Cb and Cr channels were the least among the channels.

Table 2 presents the detection accuracy and computational time results from applying PCA on RLRN with 10 and 50 dimensions. The results indicate the same behavior among different channels with the results obtained from 100D. A reduction in detection accuracy was observed for 10D compared with 100D. All detection accuracies slightly increased for 50D, except for the gray and Cr channels. Computational time in 50D also reduced compared with 100D. The results presented in Table 2 indicate a high correlation between the features after PCA application, which was proven in Figure 3.

Table 3 demonstrates the detection accuracy and computational time that resulted from combining the RLRN method with kernel PCA. These results show that gray-scale images with 50D exhibited a considerable increase in detection accuracy compared with those obtained from the original and the combined methods (88.28% versus 72.73% and 69.53%), respectively. The R, G, B, and Y channels also exhibited a substantial growth of 25% in detection accuracy. These results were anticipated from Figure 3, which indicated a low correlation among features after kernel PCA application.

However, the Cb and Cr channels did not follow the same trend and exhibited a slight decrease in 50D. The other dimensionalities were also tested for the Cb and Cr channels because of this decrease, and the results indicated that the optimal dimensionality for these two channels was in 95D with detection accuracy of 92.68%. Thus, the Cb and Cr channels contain important information focused on the first 95 dimensions. All computational time generally reduced for 10D and 50D in comparison with 100D.

**Figure 5:**
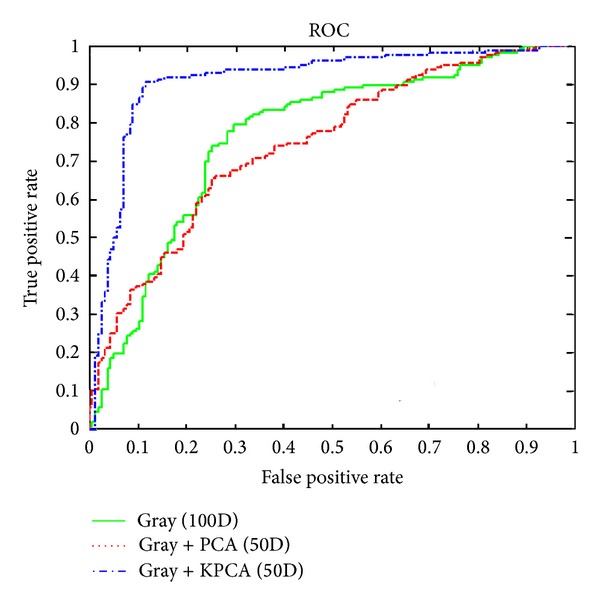
Comparison between gray-scale images in 100D, with PCA in 50D and kernel PCA in 50D.

**Figure 6:**
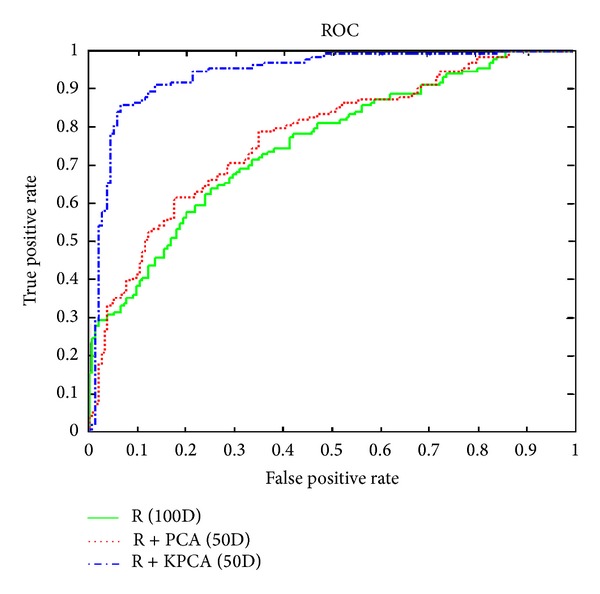
Comparison between gray-scale images in 100D, with PCA in 50D and kernel PCA in 50D.

**Figure 7:**
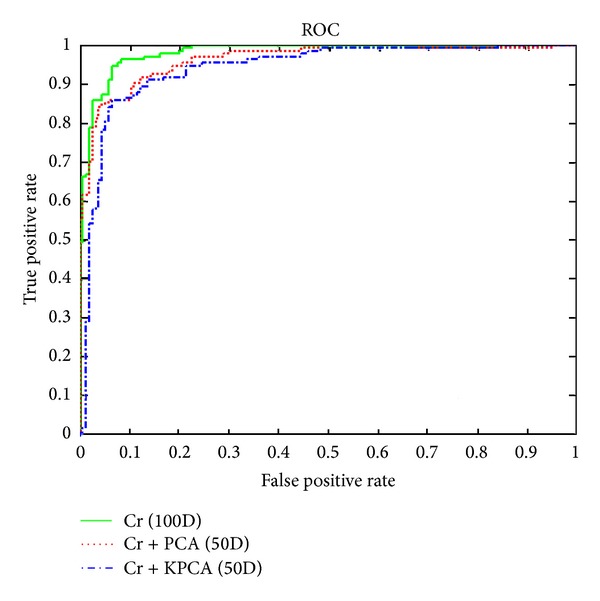
Comparison between gray-scale images in 100D, with PCA in 50D and kernel PCA in 50D.

Combining the RLRN method with kernel PCA in 50D generally exhibited the best results among the methods, which verifies the nonlinear nature of RLRN. Figures 5, 6, and 7 demonstrate the receiver operating characteristic (ROC) curves for gray-scale images and R and Cr channels, respectively. A comparison of the features extracted from the original feature extraction method in 100D was made in each figure with the extracted features from the merged ones with PCA and kernel PCA dimension reduction methods in 50D. The results obtained from 10D were ignored because of the high amount of results presented here.  Figure 5 indicates the best effect of kernel PCA on RLRN, while PCA has almost the same results with the original features.

The R, G, B, and Y channels have similar behaviors; the R channel was selected to represent the ROC in Figure 6. According to this ROC, the R, G, B, and Y channels performed better when the dimensionality of the features was reduced to 50D by applying kernel PCA.

Figure 7 shows an inverse effect of kernel PCA on the obtained features from the Cb and Cr channels. The results show that the original features performed the best, and the dimension reduction methods did not improve accuracy rate.

### 3.3. Comparison with Other Methods

A comparison of some of state-of-the-art image splicing detection methods was conducted for a comprehensive evaluation of the entire system.  Table 4 indicates the comparison between different methods and the proposed framework for gray-scale images in image dataset 1.

Table 4 shows that the accuracy rates exhibited different trends. Computational time could not be compared because it was not provided in the other methods. The best results were observed for the expanded DCT Markov + DWT Markov and expanded DCT Markov [10], which reduced to 100D by applying the SVM-RFE method (93.55% and 90.07%). The next best accuracy rate was observed for our proposed method (88.28%) with 50D. The results show that our proposed method performed the best compared with similar methods [13] with less dimensions (163D versus 50D).

Table 5 shows another comparison between the original RLRN [15] in 60D and the proposed method (RLRN + kernel PCA) in 50D on image dataset 2 (CASIA). The Gray indicated in the table was obtained by converting the colored images in image dataset 2 to gray-scale images. As previously discussed, a huge increase in detection accuracies in the R, G, B, and Y channels, as well as the gray one, was observed.

## 4. Conclusion

The literature review presented in this work showed that several image splicing detection methods were proposed. Unfortunately, many of them are unable to handle the extracted high dimensional and redundant features well. In addition, processing these features is time consuming. Therefore, this paper focused on evaluating the effectiveness of dimension reduction methods on image splicing detection methods to reduce dimensionality and computational time and remove redundant information from the features. Two dimension reduction methods (PCA: linear and kernel PCA: nonlinear) were selected and applied on an instance image splicing method (RLRN). A set of experiments were designed and tested to determine the effectiveness of these methods on RLRN. Each dimension reduction method was applied on RLRN to reduce dimensionality to 10D and 50D. The results showed that the R, G, B, and Y channels and gray-scale images performed best when merged with kernel PCA and in 50D, which verifies the nonlinear nature of the RLRN features. However, the results also demonstrate that this area of study requires further research. Other dimension reduction methods and their effects on other image splicing detection methods must be investigated, and the optimal dimension number for every image splicing detection approach must be identified.

## Figures and Tables

**Table 1:** Detection accuracy and computational time for RLRN with 100D.

	100D			
	TP (%)	TN (%)	Acc. (%)	Time (s)
R	63.91	74.68	69.30	388.43
G	57.89	71.43	64.66	402.83
B	55.64	75.32	65.48	426.27
Gray	70.51	75.00	72.75	489.72
Y	58.65	67.53	63.09	408.01
Cb	90.23	88.96	89.59	258.73
Cr	94.74	92.86	93.80	252.32

**Table 2:** Detection accuracy and computational time for RLRN with PCA in 10D and 50D.

PCA	10D				50D			
	TP (%)	TN (%)	Acc. (%)	Time (s)	TP (%)	TN (%)	Acc. (%)	Time (s)
R	60.90	73.38	67.14	447.86	66.17	74.68	70.43	302.57
G	55.63	72.08	63.86	457.46	61.65	77.92	69.79	270.28
B	67.67	69.48	68.57	504.21	64.66	72.73	68.69	293.34
Gray	67.94	75.00	71.43	462.49	65.38	73.68	69.53	297.29
Y	54.89	72.73	63.81	449.55	59.40	70.78	65.09	275.09
Cb	81.20	87.01	84.11	203.25	87.97	93.51	90.74	182.66
Cr	83.46	92.21	87.83	187.71	90.98	87.66	89.32	187.93

**Table 3:** Detection accuracy and computational time for RLRN with kernel PCA in 10D and 50D.

Kernel PCA	10D				50D			
	TP (%)	TN (%)	Acc. (%)	Time (s)	TP (%)	TN (%)	Acc. (%)	Time (s)
R	84.96	83.12	84.04	122.07	85.71	90.91	88.31	183.91
G	84.96	83.12	84.04	114.4	87.22	90.26	88.74	193.37
B	84.96	83.12	84.04	125.67	85.71	90.91	88.31	223.77
Gray	100	0	50.00	63.87	90.38	86.18	**88.28**	236.49
Y	82.71	86.36	84.54	112.78	87.22	90.26	88.74	188.15
Cb	84.96	83.12	84.04	111.67	87.81	90.91	89.36	186.7
Cr	84.96	83.12	84.04	111.58	85.71	90.91	88.31	186.62

**Table 4:** Comparison between proposed approaches and other methods.

Feature extraction methods	Dimensionality	TP (%)	TN (%)	Acc. (%)
Expanded DCT Markov [10]	100	89.92	90.21	90.07
DWT Markov [10]	100	87.58	85.39	86.50
Expanded DCT Markov + DWT Markov [10]	100	93.28	93.83	93.55
HHT + moments of characteristic functionswith wavelet decomposition [5]	110	80.25	80.03	80.15
Run length and edge statistics based model [13]	163	83.23	85.53	84.36
RLRN + kernel PCA (proposed)	**50**	**90.38**	**86.18**	**88.28**

**Table 5:** Comparison between the original RLRN [15] and the proposed method (RLRN + kernel PCA).

	RLRN [15]			RLRN + kernel PCA		
	TP (%)	TN (%)	Acc. (%)	TP (%)	TN (%)	Acc. (%)
R	56.30	83.70	70.9	85.71	90.91	88.31
G	51.80	83.20	68.50	87.22	90.26	88.74
B	57.20	83.70	71.30	85.71	90.91	88.31
Gray	60.40	81.70	71.80	86.47	90.91	88.69
Y	53.30	83.10	69.20	87.22	90.26	88.74
Cb	91.70	96.50	94.30	87.81	90.91	89.36
Cr	91.80	97.10	94.70	85.71	90.91	88.31

## Abbreviations

*n*D: *n* dimensional
RLRN: Run length run number
PCA: Principle component analysis
SVM: Support vector machine
HHT: Hilbert-Huang transform
DWT: Discrete wavelet transform
DCT: Discrete cosine transform
SVM-RFE: Support vector machine recursive feature elimination
DVMM: Digital video multimedia
CASIA: Chinese Academy of Sciences, Institute of Automation
TP: True positive
TN: True negative
Acc.: Accuracy
ROC: Receiver operating characteristics.
